# Difference of the progression of pulmonary cysts assessed by computed tomography among COPD, lymphangioleiomyomatosis, and Birt-Hogg-Dubé syndrome

**DOI:** 10.1371/journal.pone.0188771

**Published:** 2017-12-08

**Authors:** Kazunori Tobino, Toyohiro Hirai, Takeshi Johkoh, Kiminori Fujimoto, Atsushi Kawaguchi, Noriyuki Tomiyama, Kazuhisa Takahashi, Kuniaki Seyama

**Affiliations:** 1 Department of Respiratory Medicine, Iizuka Hospital, Iizuka, Fukuoka, Japan; 2 Divisions of Respiratory Medicine, Juntendo University Faculty of Medicine & Graduate School of Medicine, Bunkyo-Ku, Tokyo, Japan; 3 The Study Group of Pneumothorax and Cystic Lung Diseases, Setagaya-Ku, Tokyo, Japan; 4 Department of Respiratory Medicine, Kyoto University, Graduate School of Medicine, Sakyo-Ku, Kyoto, Japan; 5 Department of Radiology, Kinki Central Hospital of Mutual Aid Association of Public School Teachers, Itami, Hyogo, Japan; 6 Department of Radiology, Kurume University School of Medicine and Center for Diagnostic Imaging, Kurume University Hospital, Kurume, Fukuoka, Japan; 7 Center for Comprehensive Community Medicine Faculty of Medicine, Saga University, Saga, Saga, Japan; 8 Department of Radiology, Osaka University Graduate School of Medicine, Suita, Osaka, Japan; Children’s Hospital of Los Angeles, UNITED STATES

## Abstract

Many groups developed the methods to quantitatively analyze low attenuation area (LAA) on chest CT in patients with cystic lung diseases. Especially in COPD, it was reported that the cumulative size distribution of LAA clusters follows a power law characterized by the exponent D, which reflect the fractal dimension of terminal airspace geometry. We hypoyhesized that the quantitative charateristics of LAA clusters including fractal property might indicate the different features of the progression of cysts in cystic lung diseases. The aim of this study was to apply the CT image-based method of characterizing the size distribution of LAA clusters for lymphangioleiomyomatosis (LAM) and Birt-Hogg-Dubé syndrome (BHDS) to disclose their features of the progression of pulmonary cysts. 40 patients with COPD, 52 patients with LAM, and 18 patients with BHDS who had undergone CT scans at our institute between January 2002 and August 2009 were included. Differences among these diseases in the quantitative characteristics of LAA clusters {i.e., extent, number, size, fractal property, and the relationship between these quantitatives} were assessed. The Chi-sqsuare test, unpaired t-test, and one-way analyses of variance with Tukey post-hoc tests were used to compare groups, spline model with an interaction terms were used to assess the relationship between extent and number, and exponential regression model was used to assess the relationship between extent and size. Statistically significant differences separated the three diseases in extent and number (P < 0.001). Number was significantly correlated with extent in COPD (P < 0.001), but was not so in LAM and BHDS when extent exceeded 11.5% and 20.8%, respectively. Size was significantly correlated with extent in COPD and LAM (P < 0.001), but was not so in BHDS. The percentage of CT images with fractal property was higher in COPD than that in LAM and BHDS (95.8%, 92.9% and 63.0%, respectively). In conclusion, our study has demonstrated for the first time the different characteristics of the size distribution of LAA clusters among COPD, LAM and BHDS, and indicated that this method is useful for exploration of the pathophysiology in cystic lung diseases.

## Introduction

Both emphysema and pulmonary cysts are recognized as low attenuation areas (LAA) on chest computed tomography (CT). Recently, many groups developed the methods to quantitatively analyze LAA on chest CT in patients with cystic lung diseases {i.e., chronic obstructive pulmonary disease (COPD), lymphangioleiomyomatosis (LAM) and Birt-Hogg-Dubé syndrome (BHDS)}, and they reported the correlation between those quantitatives and pulmonary function or symptoms [1–3].

Especially in COPD, Mishima et al. analyzed the numbers and sizes of LAA clusters and found that the cumulative size distribution of LAA clusters follows a power law characterized by the exponent D [4]. The values of D reflect the fractal dimension of terminal airspace geometry and sensitively detect alveolar tissue destruction. Thereafter, the simulation analysis by Suki et. al. and the longitudinal CT study by Tanabe et. al. revealed that the fractal property of LAA clusters on chest CT in COPD was explained by applying a model of mechanical force-based destruction in emphysema development [5–7]. Accordingly, the analysis of size distribution of LAA clusters on chest CT is thought to be a very useful method for consideration of pathogenesis and pathophysiologies in COPD.

To our knowledge, there is no study that examined the characteristics of size distribution of LAA clusters on chest CT in cystic lung diseases other than COPD, in which the pathogenesis of pulmonary cysts are distinctly different from that of COPD. We hypoyhesized that the quantitative charateristics of LAA clusters including fractal property would be quite different among cystic lung diseases, and might indicate their features of the progression of pulmonary cysts. Therefore, the purpose of the present study is to apply this CT image-based method for LAM and BHDS to disclose their features of the progression of pulmonary cysts.

## Materials and methods

### Study population

This retrospective study was approved by the ethics committee of Juntendo Hospital (JIRB21-134), and the requirement to obtain informed consent was waived. This study included consecutive patients with COPD, LAM, and BHDS who had undergone thin-section CT scans at our institute between January 2002 and August 2009. COPD was diagnosed according to the criteria of the Global Initiative for Obstructive Lung Disease Workshop Report [8]. LAM was diagnosed from tissue biopsies or characteristic clinical pictures (recurrent pneumothorax and/or chylous pleural effusion) and CT findings (diffusely scattered thin-walled pulmonary cysts). Tuberous sclerosis complex-associated LAM (TSC-LAM) was diagnosed by means of established clinical criteria [9]. The diagnosis of BHDS was obtained with the *FLCN* gene mutation analysis [10]. Patients who had pneumothorax or pleural effusion were excluded.

In COPD, consecutive 40 patients were included {38 male with mean age of 66.7 years (age range, 43–81 years), and 2 female with mean age of 74.0 years (age range, 73–75)} without exclusions of patients. In LAM, after the exclusion of eight patients with pneumothorax and six patients with hydrothorax, 52 patients were finally enrolled {all female with mean age of 36.4 years (age range, 43–81 years)}. Three patients were tuberous sclerosis complex-associated LAM (TSC-LAM). In BHDS, after the exclusion of four patients with pneumothorax, 18 patients were finally enrolled {four male with mean age of 35.3 years (age range, 33–38 years), and 14 female with mean age of 45.6 years (age range, 26–80)}.

### Thin-section CT techniques

All patients underwent thin-section CT with an 8-detector row CT scanner or 64-detector row CT scanner (Aquilion 8 or Aquilion 64; Toshiba Medical, Tokyo, Japan) with a 2-mm slice thickness and scanning parameters of 120 kVp, 150 mAs and a field of view of 320 mm. No contrast media were used. During the scan, the patients held their breath after a deep inspiration in the supine position. Each CT image was composed of a 512 × 512 matrix of numeric data (CT numbers) in Hounsfield units (HU) reconstructed using a lung algorithm (FC83). The area of 1 pixel is 0.39 mm^2^.

### Analysis of LAA clusters

Three slices from each patient were analyzed: the upper slice taken 1cm above the upper margin of the aortic arch; the middle slice taken 1cm below the carina; and the lower slice taken 1cm above the top of the diaphragm. Each lung zone were thought to have different degrees of disease progression, therefore, all of the derived values from each lung slice were used for the evaluation as an independent value representing disease state (i.e., three data were obtained from one patient).

**Fig 1:**
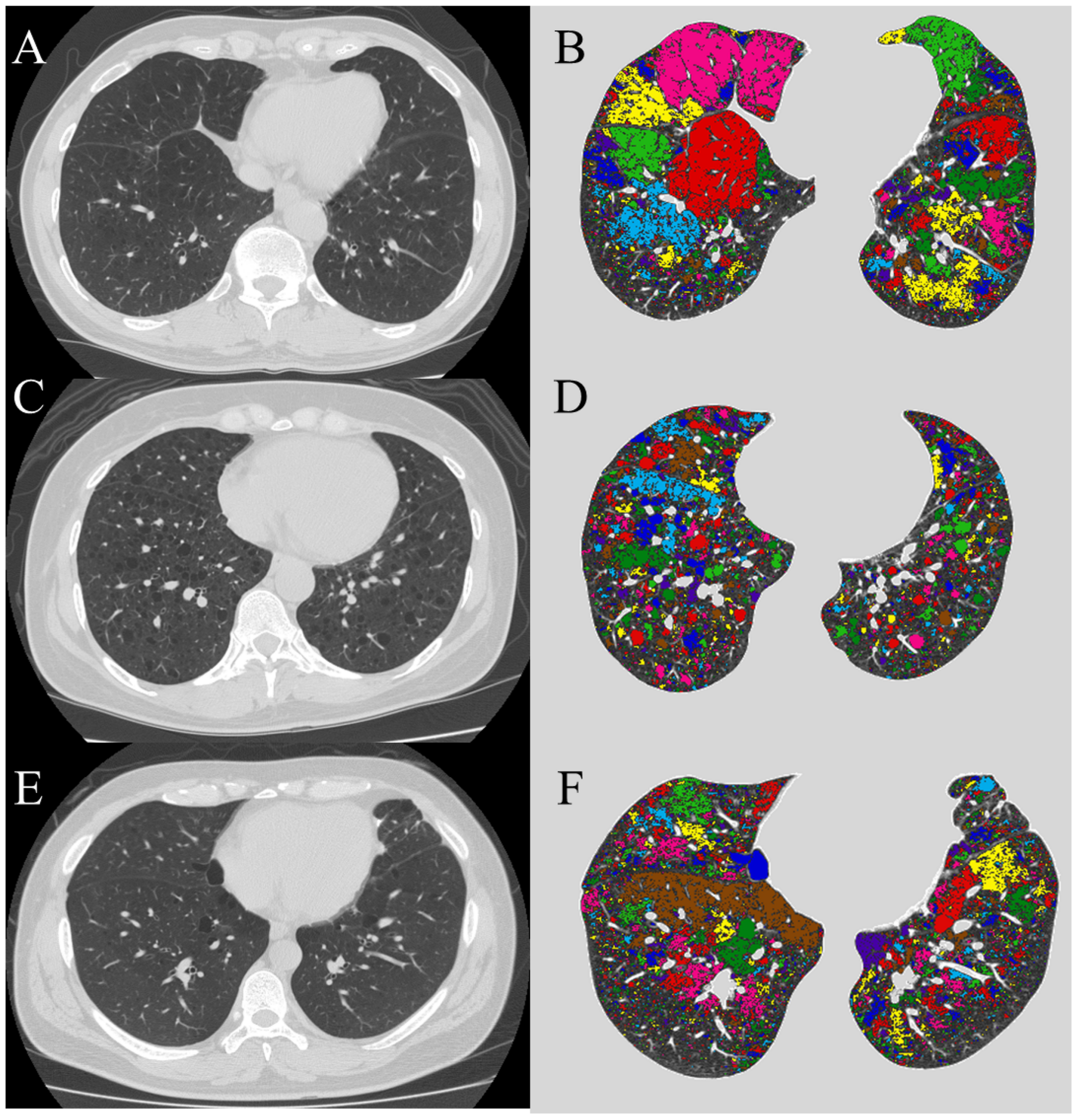
Analysis of LAA clusters on CT images. Original CT image of the lower lung field, and the same image as in original image but the individual cluster comprising contiguous LAA is shown in contrasting colors in a representative patient with COPD (A and B), LAM (C and D) and BHDS (E and F). The lung field was identified from the rest of the image, and the lumina of airways were excluded. In COPD, LAA also exists in seemingly normal region, and contiguous LAA regions (colored area) fuse and become large. On the other hand, in LAM and BHDS, LAAs are few in seemingly normal areas, and the contiguous LAA regions (colored area) seems to be independent. BHDS: Birt-Hogg-Dubé syndrome; COPD: chronic obstructive pulmonary disease; LAA: low attenuation areas; LAM: lymphangioleiomyomatosis.

The percentage of LAA (LAA%) was calculated automatically according to the method reported previously (1,4). We defined lung fields as areas with CT numbers less than −200 HU, whereas the cut-off level between LAA and normal lung density was −960 HU (Fig 1) [11]. The number and mean size of LAA clusters were quantitatively assesed automaltically (LAA_n and LAA_s, respectively).

#### Fractal analysis

**Fig 2:**
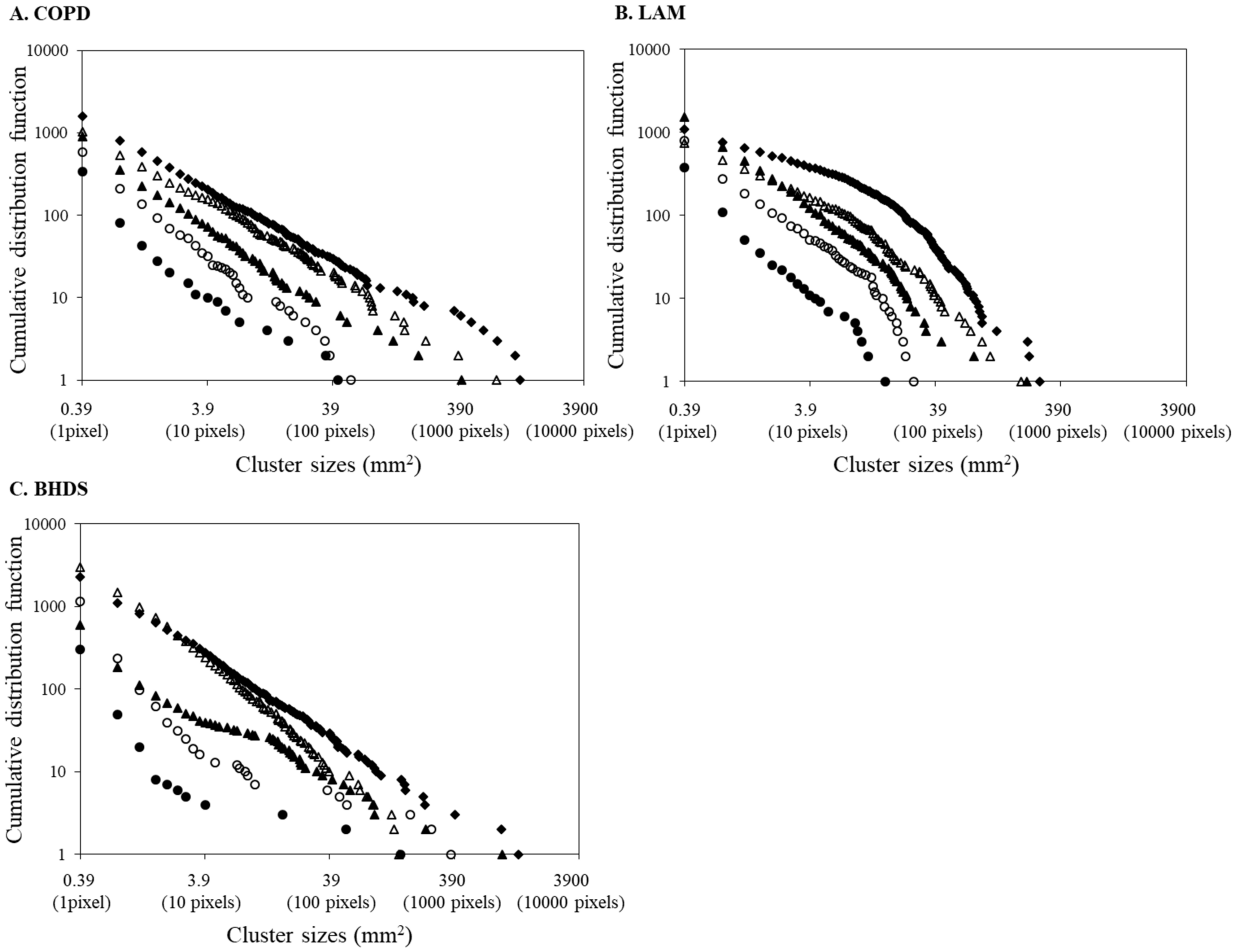
Log–log plots of representative cumulative frequency distributions of the size of LAA clusters. In subjects with COPD (A), the five lung slices analyzed had LAA% values of 2.2 (●), 5.0 (〇), 15.2 (▲), 28.0 (Δ), and 42.1 (◆). In these five slices, *r*^*2*^ has a value of 0.96, 0.98, 0.99, 0.98, and 0.99, respectively. The corresponding D values are 0.97, 1.12, 0.98, 0.96, and 0.83, respectively. In subjects with LAM (B), the five lung slices analyzed had LAA% values of 2.0 (●), 6.7 (〇), 16.7 (▲), 26.9 (Δ), and 40.9 (◆). In these five slices, *r*^*2*^ has a value of 0.96, 0.93, 0.98, 0.95, and 0.89, respectively. The corresponding D values are 1.24, 1.34, 1.27, 1.11, and 1.11, respectively. In subjects with BHDS (C), the five lung slices analyzed had LAA% values of 2.0 (●), 5.7 (〇), 15.9 (▲), 24.6 (Δ), and 40.2 (◆). In these five slices, *r*^*2*^ has a value of 0.72, 0.84, 0.97, 0.99, and 1.0, respectively. The corresponding D values are 0.74, 0.78, 0.76, 1.30, and 0.97, respectively. BHDS: Birt-Hogg-Dubé syndrome; COPD: chronic obstructive pulmonary disease; LAA: low attenuation areas; LAA%: percentage of lung field occupied by low attenuation areas; LAM: lymphangioleiomyomatosis.

Fractals are self-similar structures characterized by power-law functions and noninteger dimensions (fractal dimension) (PNAS Sato). To assess the fractal properties of the distributions of LAA sizes, first, cumulative frequency distributions of the LAA_s in each CT image were plotted with a logarithmic scale (log-log plot). Fig 2 demonstrates that the cumulative frequency distributions of LAA_s decrease on log–log plots. The slopes of plots vary between subjects and as a function of LAA%. Next, power law functions were created to fit the slopes of the plots: Y = K * X^-D^ (Y, the cumulative frequency distribution; X, LAA_s). Values of coefficient of determination (*r*^*2*^) were taken to indicate the goodness-of-fit of the power law functions [4], and it was defined as having fractal property with an *r*^*2*^ value of 0.9 or more.

The differences among the three diseases about quantitative characteristics of LAA clusters, characteristics of relationships between LAA% and LAA_n or LAA_s, and fractal property (*r*^*2*^) were examined. The relationship between the LAA_n and LAA% was modeled by regression techniques using a spline model with an interaction terms [12]. First, LAA_n was set to objective variable, and LAA% and disease group (i.e., LAM, BHDS, or COPD) were set to explaining variables. Next, the number and location of knots were determined objectively, and Akaike’s Information Criterion (AIC) was used to assess the goodness of model fitness. Therefore, four knots were made at 20, 40, 60, and 80 percentiles of LAA%, and five intervals of LAA% were made as follows; LAA% between 0% and 4.2% (interval I), 4.2 and 11.5% (interval II), 11.5% and 20.8% (interval III), 20.8% and 34.2% (interval IV), and LAA% of 34.2% or more (interval V).

### Statistical analysis

The Chi-square test was used for qualitative variables and one-way analyses of variance with Tukey post-hoc tests were used for quantitative variables to compare groups. The unpaired t-test was used to compare two groups. All statistical analyses were performed using SPSS software (version 16.0, SPSS Inc., Chicago). Data were expressed as mean ± SD, and values of p < 0.05 were regarded as statistically significant.

## Results

### Patients characteristics

Patients characteristics are presented in Table 1. There were statistical significant differences among the three groups in sex ratio, age, height and smoking history.

**Table 1:** Patients characteristics.

	COPD	LAM	BHDS
**Number of patients**	40	52	18
**Male/Femal**	38/2	0/52^‡^	4/14^†^^‡^
**Age (y)**	67.1 ± 8.4	36.4 ± 8.5^†^	43.3 ± 13.8^†^^‡^
**Height (cm)**	164.8 ± 8.2	159.3 ± 4.5^†^	157.6 ± 8.6^†^
**Smoking history (pack-yearrs)**	59.5 ± 27.8	2.0 ± 5.7^†^	3.7 ± 8.8^†^

BHDS: Birt-Hogg-Dubé syndrome; COPD: chronic obstructive pulmonary disease; LAM: lymphangioleiomyomatosis.

Values are expressed as means ± SD where applicable.

^†^ *p* < 0.001 compared with COPD.

^‡^ *p* < 0.01 compared with LAM.

### Analysis of LAA clusters

**Fig 3:**
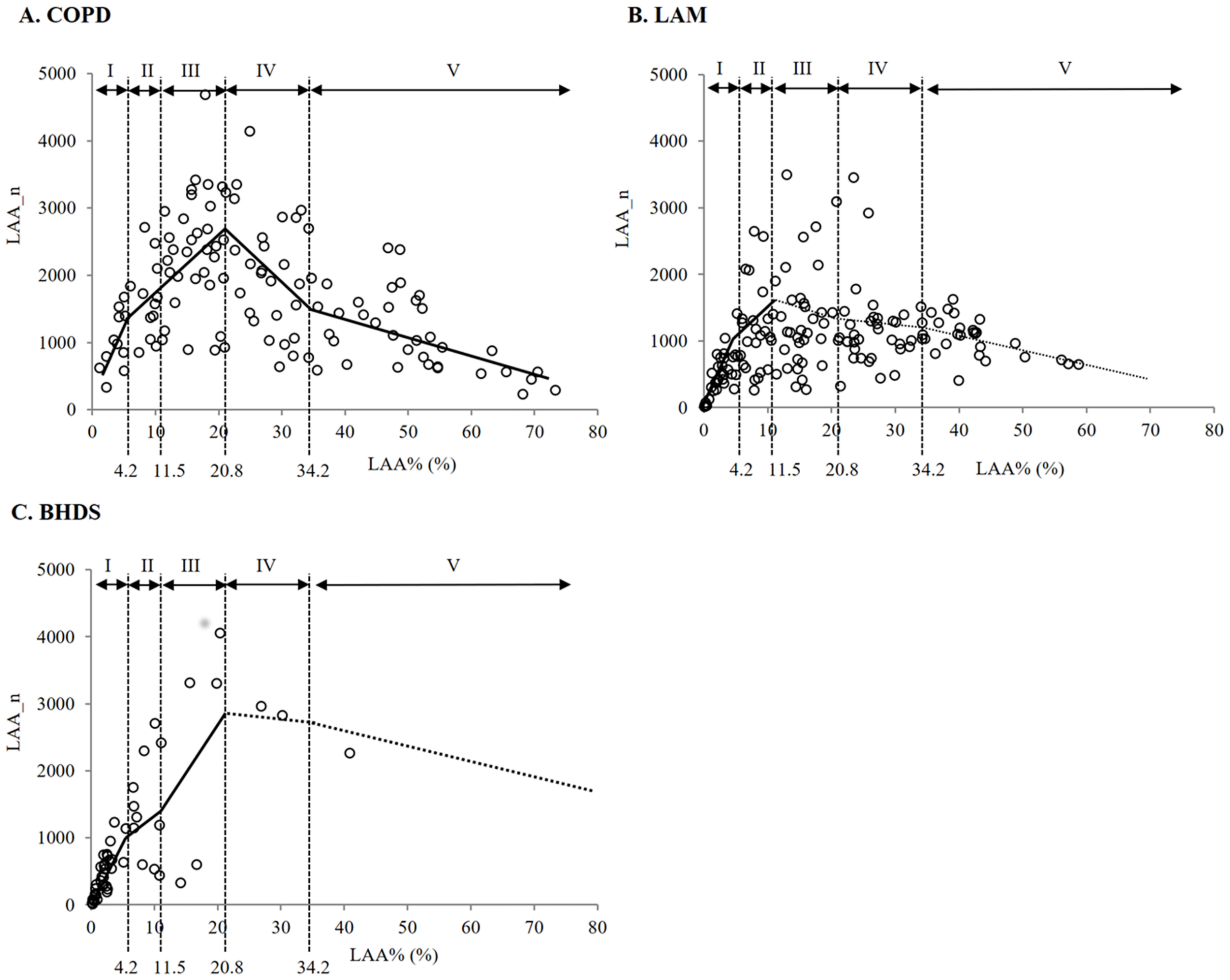
The relationships between LAA_n and LAA% in all lung fields. Solid and dashed line shows that the correlation is statistically significant and non-significant with the linear regression with spline models, respectively. A: In COPD, LAA_n increased once and thereafter decreased with increase of LAA%. In all intervals, LAA_n-LAA% relationships were statistically significant. B: In LAM, LAA_n increased with increase of LAA% from 0% to 11.5%, and thereafter LAA%- LAA_n relationship was not statistically significant. C: In BHDS, LAA_n increased with increase of LAA% from 0% to 20.8%, and thereafter LAA_n-LAA% relationship was not statistically significant. LAA%: percentage of lung field occupied by low attenuation areas; LAA_n: number of LAA cluster.

**Fig 4:**
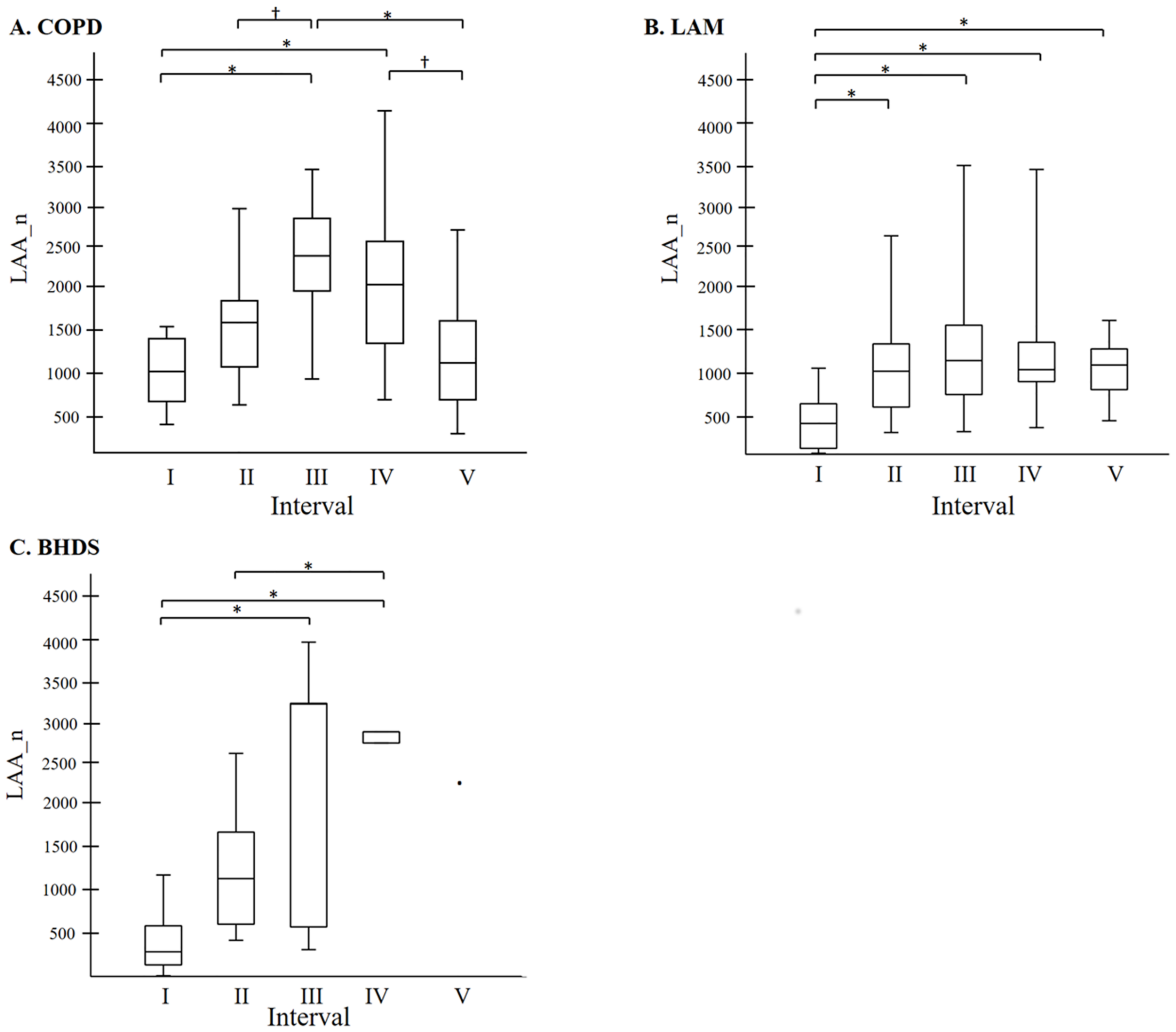
The comparison of LAA_n among five LAA%-intervals. A: In COPD, interval III was the highest and intervals I and II were lower. B: In LAM, interval I was the lowest and there was no statistically significant difference among intervals II to V. C. In BHDS, intervals III and IV were higher than the other intervals. The line across the box indicates at the median. The bottom of the box is at 25th percentile, and the top is at 75th percentile value. The minimal and maximal values are shown with short horizontal lines ("whiskers"). LAA%: percentage of lung field occupied by low attenuation areas; LAA_n: number of LAA cluster * p < 0.05; † p < 0.01.

The results of the quantitative analyses of LAA clusters (LAA%, LAA_n, and LAA_s) are shown in Table 2. Significant differences separated the three groups in LAA% and LAA_n. Among the three diseases, BHDS had the lowest value in LAA% and LAA_s, while COPD had the highest value in LAA%, LAA_n, and LAA_s in the upper lung field. Fig 3 shows the relationship between LAA_n and LAA% modeled by regression techniques using a spline model in each disease. In COPD, LAA_n increased once and thereafter decreased with the increase of LAA%. In all intervals, LAA_n-LAA% relationships were statistically significant. In LAM, LAA_n increased with the increase of LAA% from 0% to 11.5%, and thereafter LAA_n-LAA% relationship was not statistically significant. In BHDS, LAA_n increased with the increase of LAA% from 0% to 20.8%, and thereafter LAA_n-LAA% relationship was not statistically significant because only three images had LAA% higher than 20.8%. Table 3 shows the estimated slope of the correlation between LAA_n and LAA% in each interval of LAA% from linear regression with spline models. The comparison of LAA_n among five LAA%-intervals in each disease (Fig 4) can confirm the tendency of LAA_n-LAA% relationships demonstrated in Fig 3.

**Table 2:** Results of the quantitative analysis of LAAs.

	COPD	LAM	BHDS
LAA% (%)			
Middle lung field	30.9 ± 17.9	16.3 ± 13.9^‡^	4.2 ± 6.5^‡^^§^
Upper lung field	24.2 ± 15.0	21.1 ± 15.2	5.2 ± 7.3^‡^
Lower lung field	27.2 ± 20.0	18.5 ± 13.3^‡^	9.9 ± 10.1^‡^**
All images	27.4 ± 17.8	18.7 ± 14.2^‡^	6.4 ± 8.3^‡^
LAA_n			
Upper lung field	1191.2 ± 554.5	795.4 ± 474.7^††^	790.9 ± 942.0^†^
Middle lung field	1994.6 ± 863.5	1081.3 ± 621.1^‡^	968.7 ± 976.5^‡^
Lower lung field	1969.6 ± 980.5	1217.5 ± 730.2^‡^	1015.3 ± 1110.8^‡^
All images	1718.4 ± 894.7	1031.4 ± 638.5^‡^	925.0 ± 997.9^‡^
LAA_s (mm^2^)			
Upper lung field	6.8 ± 8.9	1.4 ± 1.2^†^	0.4 ± 0.3^††^
Middle lung field	3.9 ± 4.4	0.7 ± 7.3	1.1 ± 0.6^§^
Lower lung field	6.3 ± 12.0	11.2 ± 4.0	2.3 ± 2.1
All images	5.7 ± 0.8	4.2 ± 4.3	1.4 ± 1.4^‡^**

BHDS: Birt-Hogg-Dubé syndrome; COPD: chronic obstructive pulmonary disease; LAA: low attenuation areas; LAA%: percentage of lung field occupied by low attenuation areas; LAA_n: number of LAA cluster; LAA_s: mean size of LAA cluster; LAM: lymphangioleiomyomatosis

Values are expressed as means ± SD where applicable.

^†^ p < 0.05 compared with COPD.

^††^ p < 0.01 compared with COPD.

^‡^ p < 0.001 compared with COPD.

** p < 0.05 compared with LAM.

^§^ p < 0.01 compared with LAM.

**Table 3:** The estimated slope of the correlation between LAA% and LAA_n for linear regression with spline models in each interval of LAA%.

	COPD		LAM		BHDS	
Intervals of LAA%	Estimated slope	P value	Estimated slope	P value	Estimated slope	P value
I (0–4.236)	208.7	< 0.0001	208.7	< 0.0001	208.7	< 0.0001
II (4.236–11.470)	78.6	0.0008	78.6	0.0008	78.6	0.0008
III (11.470–20.772)	82.8	0.0001	-13.5	0.5254	150.5	< 0.0001
IV (20.772–34.208)	-79.2	< 0.0001	-6.7	0.686	-11.4	0.5985
V (34.208 –)	-27.9	0.0005	-20.7	0.1751	-25.5	0.4204

BHDS: Birt-Hogg-Dubé syndrome; COPD: chronic obstructive pulmonary disease; LAA%: percentage of lung field occupied by low attenuation areas

**Fig 5:**
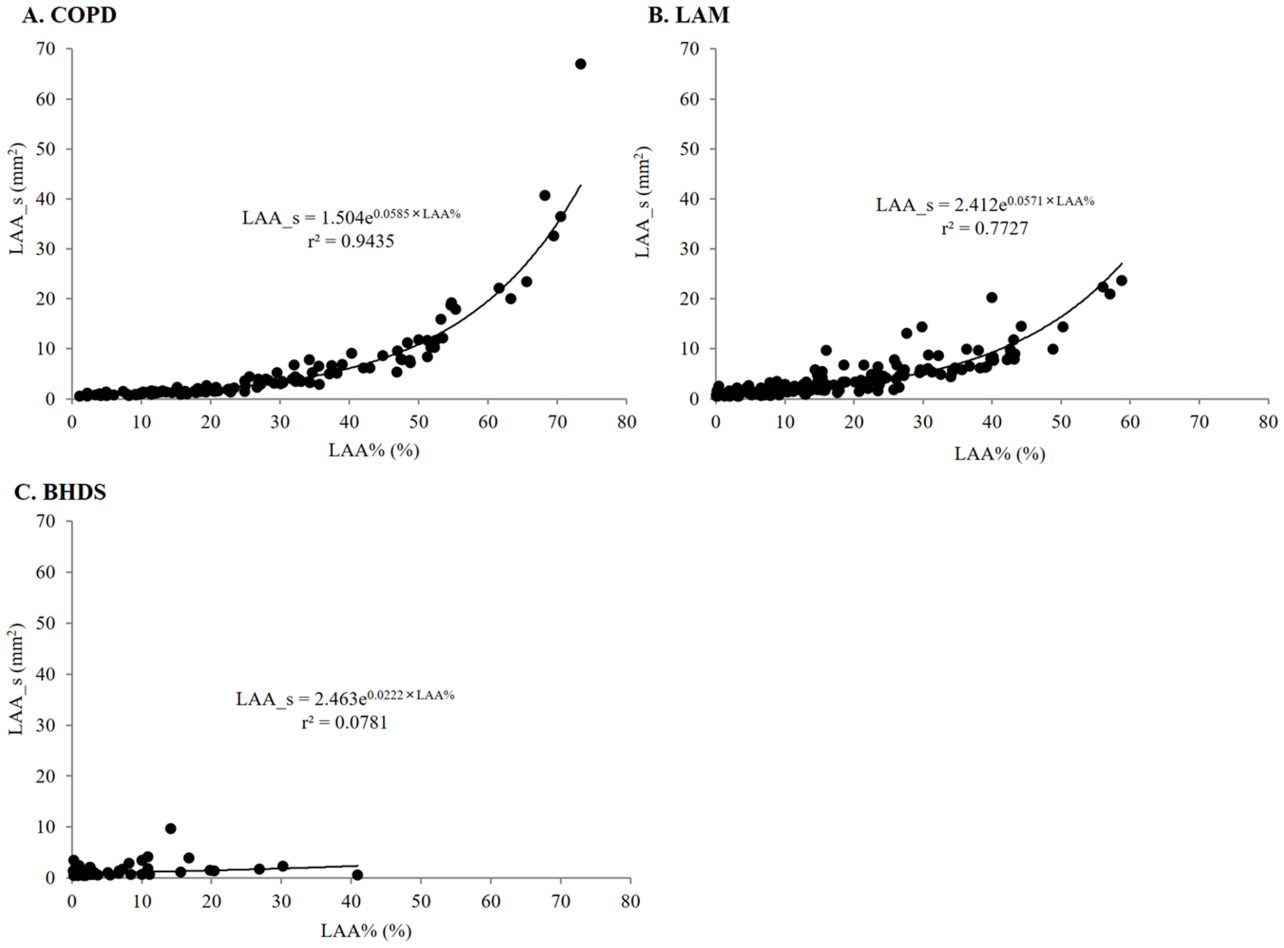
The relationships between LAA_s and LAA% in all lung fields. A and B: In COPD and LAM, LAA_s increased exponentially with LAA%. C: In BHDS, LAA_s did not correlate with LAA%. BHDS: Birt-Hogg-Dubé syndrome; COPD: chronic obstructive pulmonary disease; LAA%: percentage of lung field occupied by low attenuation areas; LAA_s: mean size of LAA cluster; LAM: lymphangioleiomyomatosis.

**Fig 6:**
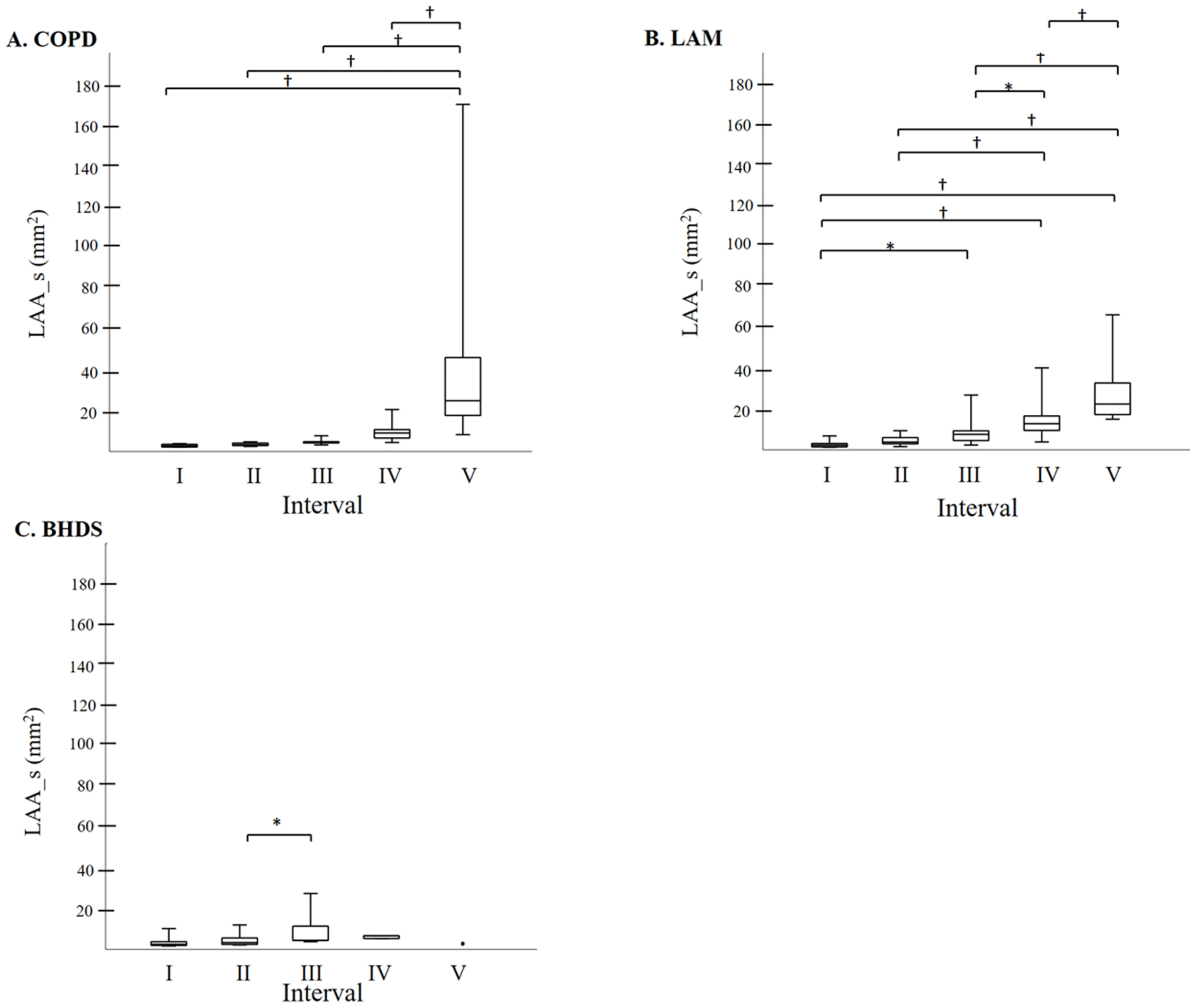
The comparison of LAA_s among five LAA%-intervals. A: In COPD, interval V was the highest and there was no statistically significant difference among intervals I to IV. B: In LAM, intervals I and II were lower and thereafter LAA_s increased step by step from intervals III to V. C: In BHDS, interval III was slightly higher than II, and there was no significant difference among the other intervals. The line across the box indicates the median. The bottom of the box is at 25th percentile, and the top is at 75th percentile value. The minimal and maximal values are shown with short horizontal lines ("whiskers"). BHDS: Birt-Hogg-Dubé syndrome; COPD: chronic obstructive pulmonary disease; LAA%: percentage of lung field occupied by low attenuation areas; LAA_s: mean size of LAA cluster; LAM: lymphangioleiomyomatosis * p < 0.05, † p < 0.01.

Fig 5 shows the relationship between LAA_s and LAA% in each disease. In COPD and LAM, LAA_s can be expressed as an exponential function of LAA%, and the coefficient of determination was higher in COPD than that in LAM (0.9435 and 0.7727, respectively). In BHDS, there was no significant relationship between LAA_s and LAA%. The comparison of LAA_s among five LAA%-intervals in each disease (Fig 6) can confirm the tendency of LAA_s-LAA% relationships demonstrated in Fig 5. Finally, based on the previous report [3], we categorized the size of LAA cluster into three groups as follows: small (< 10pixels = 3.9 mm^2^), medium (10 – 100pixels = 3.9–39 mm^2^), and large (> 100 pixels = 39 mm^2^), and calculated the percentage of each size-catergorized LAA_n in the entire LAA_n of each CT image. In LAM, the increase of medium-sized LAA_n percentage stands out more than other diseases (online supplementary S1 and S2 Figs for further details).

**Fig 7:**
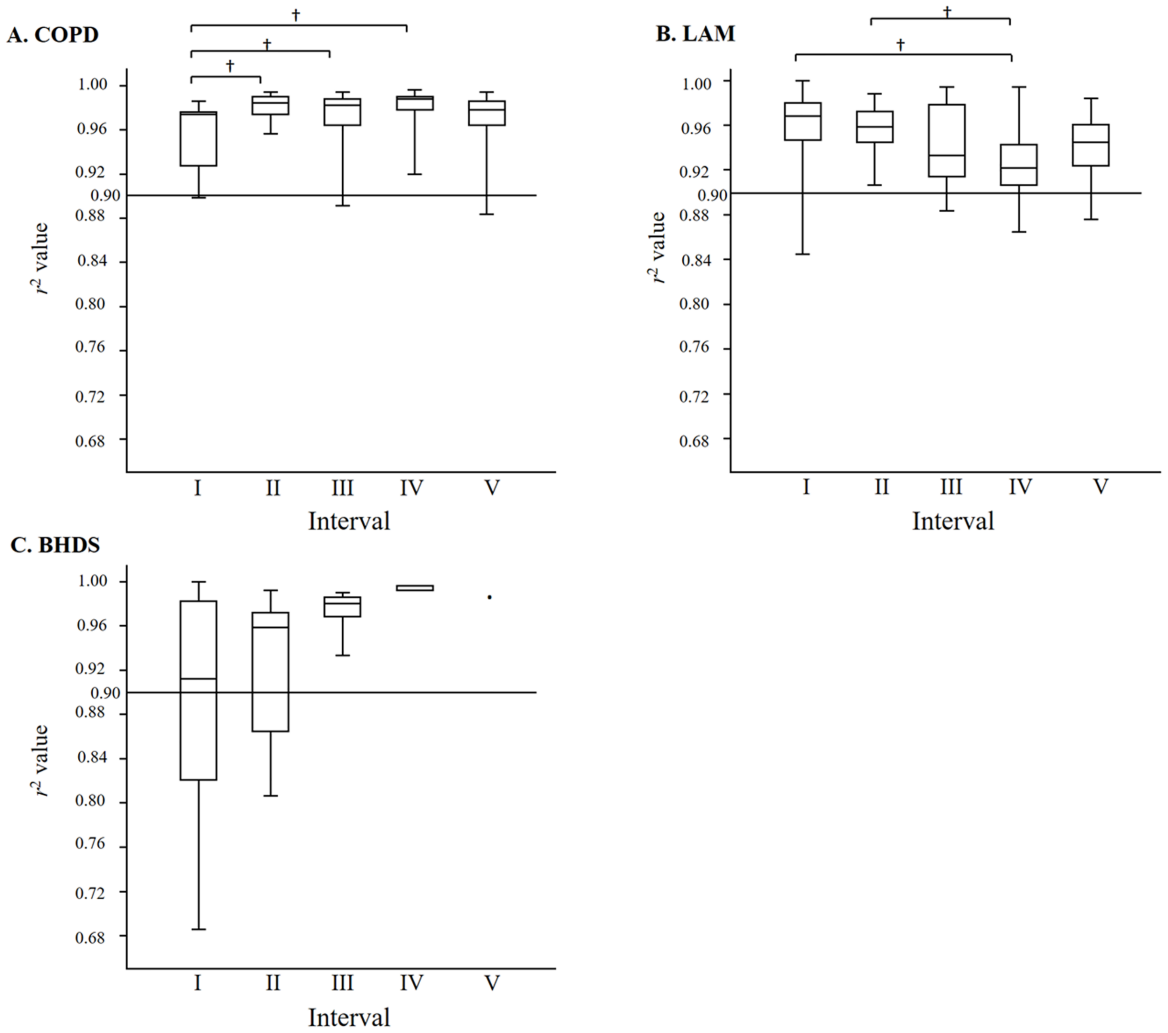
The comparison of *r*^2^ value among five LAA%-intervals. A: In COPD, interval I was lower than II, III and IV, and there was no statistically significant difference among intervals II to V. B: In LAM, interval IV was lower than intervals I and II. The *r*^2^ value seemed to decrease step by step from intervals I to IV. C. In BHDS, there was no statistically significant difference among all intervals, however, *r*^2^ value seemed to increase step by step from intervals I to V. The line across the box indicates at the median. The bottom of the box is at 25th percentile, and the top is at 75th percentile value. The minimal and maximal values are shown with short horizontal lines ("whiskers"). The horizontal line at *r*^2^ = 0.90 in each panels A to C indicates the definition of fractal property. The figures above intervals in each panel indicate the percentage of CT images with fractal propety. BHDS: Birt-Hogg-Dubé syndrome; COPD: chronic obstructive pulmonary disease; LAA%: percentage of lung field occupied by low attenuation areas; LAM: lymphangioleiomyomatosis; *r*^*2*^: values of coefficient of determination which indicate the goodness-of-fit of the power law * p < 0.05, † p < 0.01.

Fig 2 shows log–log plot of representative cumulative frequency distributions of the size of LAA clusters in each disease. In COPD, the cumulative frequency distribution can be significantly described by a power law of the size of LAA clusters, compared with LAM and BHDS. The mean values of coefficient of determination (*r*^*2*^) in COPD, LAM, and BHDS were 0.975, 0.944, and 0.912, respectively. The *r*^*2*^ value in COPD was significantly higher than that in LAM and BHDS (*p* < 0.001), and the *r*^*2*^ value in LAM was higher than that in BHDS (*p* < 0.001). Fig 7 shows the comparions of *r*^2^ value among five LAA%-intervals in each disease. In COPD, interval I was lower than II, III and IV, and there was no statistically significant difference among interval II to V. The median *r*^2^ value of each LAA%-interval was higher than 0.96. In LAM, interval IV was lower than interval I and II, and the *r*^2^ value seemed to decrease step by step from interval I to IV. In BHDS, there was no statistically significant difference among all intervals, however, the *r*^2^ value seemed to increase step by step from interval I to V. The percentages of the CT images that have fractal property (defined as *r*^*2*^ value more than 0.9) are 95.8%, 92.9%, and 63.0% in COPD, LAM, and BHDS, respectively.

## Discussion

Our study has demonstrated the different characteristics of the size distribution of LAA clusters among COPD, LAM and BHDS.

In COPD, emphysema is thought to result from chronic inflammation followed by increased alveolar wall cell death and/or failure of alveolar wall maintenance [13,14]. Previous study assessed the cumulative size distributions of LAA clusters on chest CT and reported that emphysematous lung had fractal property regardless of LAA% value [4]. Also in our COPD patients, most of the CT images (95.8%) had fractal property regardless of LAA% value. Understandably, both of the LAA_n-LAA% and LAA_s-LAA% relationships were the same as previous reports (1). This phenomenon is thought to be due to the developmental process of emphysema which is explained by applying “elastic spring network model” or “model of mechanical force-based destruction” [4,6–7]. These models mean that alveolar wall rupture is not uniform in the lung, and might be more severe in local regions of relatively advanced emphysema.

LAM is thought to be a neoplastic disorder, and metastatic pulmonary lesions induce protease-antiprotease imbalance in local milieus leading to connective tissue matrix degradation and cyst formation [15–17]. This putative mechanism is analogous to one of the mechanisms for emphysema, but the substantial difference in both diseases is its extent in the parenchyma. The protease-antiprotease imbalance is diffuse in COPD with varying intensity, whereas the imbalance is likely to be limited to the sites of LAM cell infiltration. Moreover, LAM cell infiltration of distal airways which leads to airway narrowing and air trapping may also have a role in cyst formation [18]. In our LAM patients, the characteristics of LAA_n-LAA% relationships is quite different from those in COPD. Moreover, the percentage of CT images with fractal property is lower than that in COPD (92.9% in LAM and 95.8% in COPD), and tends to decrease when LAA% increases (Fig 7). These results can be explained by the fact that medium-sized LAA clusters increase as LAA% increases. From these analyses, it seems that “mechanical force-based destruction model” can not fully explain the way of progression of pulmonary cysts in LAM. Other than pulmonary cysts, LAM cells induce lymphatic vessel hyperplasia and lymphatic obstruction resulting in pulmonary lymphedema, and also involve pulmonary vessels resulting in pulmonary hemorrhage [17, 19–21]. These pathological changes may play a key role in preventing expansion and fusion of cysts.

BHDS is a rare autosomal dominant inherited genodermatosis, and the *FLCN* gene located in chromosome 17p11.2 has recently been identified to be defective [22–24]. The mechanism for the progression of pulmonary cysts in BHDS remains unclear, but several hypotheses have been presented. Graham et al. postulated that folliculin may play a role in lung growth [25]. Painter et al. reported that pulmonary cysts may develop from inflammation caused by macrophages or fibroblasts in which folliculin mRNA is strongly expressed [26]. Recently, Furuya et al. reported that dysregulation of the mTOR pathway may induce cyst formation through proliferation of alveolar type II (ATII) cells [27], while Kumasaka et al. postulated that *FLCN* mutation may result in abnormalities at the alveolar-septal junction with few inflammatory changes [28]. And more recently, Goncharova et al. reported that pulmonary cysts in BHDS may result from an underlying defect in ATII survival, attributable to FLCN regulation of the E-cadherin-LKB1-AMPK axis [29]. In our BHDS patients, both of LAA_n-LAA% and LAA_s-LAA% relationships were quite different from those in COPD and LAM. The percentage of CT images with fractal property is very low (63.0%), and tends to increase as LAA% increases. These results can be explained by the fact that very large-sized LAA clusters already exist in CT images with low LAA%, and the number of small to medium-sized LAA clusters increase as LAA% increases. These analyses show that the mechanism of the progression of pulmonary cysts in BHDS has quite different characteristics from those in COPD and LAM. Therefore, the hypotheses of Graham, Kumasaka, and Goncharova may accord with the results of our image analyses.

Our study may have several limitations. First, this is a retrospective study, and the number of patients with BHDS included is relatively small. Second, this is a cross-sectional study. In patients with BHDS, it was reported that there was no significant correlation between pulmonary function and LAA% (3), and LAA% may not represent disease severity and/or activity. Therefore, longitudinal analysis is needed.

In conclusion, our study has demonstrated for the first time the different characteristics of the size distribution of LAA clusters among COPD, LAM and BHDS. These results seem to represent the difference in the mechanism of the progression of pulmonary cysts, and a longitudinal study is necessary to confirm it. Our study indicates that quantitative analysis of LAA clusters on CT images is useful for exploration of the pathophysiology in cystic lung diseases.

## Supporting information

S1 Fig The scatterplots of LAA_n% (Y) against LAA% (X) in each diseases (A: COPD, B: LAM, and C: BHDS). LAA_n% means the ratio (%) of each size-catergorized LAA_n {i.e., small (●), medium (○), and large (▲)} to the entire LAA_n of the CT image. In LAM, the increase of medium LAA_n% stands out more than other diseases.
BHDS: Birt-Hogg-Dubé syndrome; COPD: chronic obstructive pulmonary disease; LAA%: percentage of lung field occupied by low attenuation areas; LAA_n: number of LAA cluster; LAM: lymphangioleiomyomatosis.
(TIF)

S2 Fig The comparison of mean medium LAA_n% value of COPD (●) and LAM (■) in each interval of LAA%. LAM is significantly higher than COPD in the interval II to V. The standard deviation values are shown with short horizontal lines.
COPD: chronic obstructive pulmonary disease; LAA%: percentage of lung field occupied by low attenuation areas; LAA_n: number of LAA cluster; LAA_n%: the ratio (%) of each size-catergorized LAA_n {i.e., small (●), medium (○), and large (▲)} to the entire LAA_n of the CT image; LAM: lymphangioleiomyomatosis
* p < 0.01.
(TIF)

## Abbreviations

BHDS: Birt-Hogg-Dubé syndrome
COPD: chronic obstructive pulmonary disease
LAA: low attenuation areas
LAA%: percentage of lung field occupied by low attenuation areas
LAA_n: number of LAA cluster
LAA_s: mean size of LAA cluster
LAM: lymphangioleiomyomatosis

## References

[1] Sakai N, Mishima M, Nishimura K, Itoh H, Kuno K. An automated method to assess the distribution of low attenuation areas on chest CT scans in chronic pulmonary emphysema patients. Chest 1994;106(5):1319–25. 7956377 10.1378/chest.106.5.1319

[2] Avila NA, Kelly JA, Dwyer AJ, Johnson DL, Jones EC, Moss J. Lymphangioleiomyomatosis: correlation of qualitative and quantitative thin-section CT with pulmonary function tests and assessment of dependence on pleurodesis. Radiology. 2002;223(1):189–97. https://doi.org/10.1148/radiol.2231010315 11930066 10.1148/radiol.2231010315

[3] Tobino K, Hirai T, Johkoh T, Kurihara M, Fujimoto K, Tomiyama N, et al Differentiation between Birt-Hogg-Dubé syndrome and lymphangioleiomyomatosis: quantitative analysis of pulmonary cysts on computed tomography of the chest in 66 females. Eur J Radiol. 2012;81(6):1340–6. https://doi.org/10.1016/j.ejrad.2011.03.039 21550193 10.1016/j.ejrad.2011.03.039

[4] Mishima M, Hirai T, Itoh H, Nakano Y, Sakai H, Muro S, et al Complexity of terminal airspace geometry assessed by lung computed tomography in normal subjects and patients with chronic obstructive pulmonary disease. Proc Natl Acad Sci USA 1999;96(16):8829–34. 10430855 10.1073/pnas.96.16.8829 PMC17692

[5] Winkler T, Suki B. Emergent structure-function relations in emphysema and asthma. Crit Rev Biomed Eng 2011;39(4):263–280. 22011233 10.1615/critrevbiomedeng.v39.i4.20 PMC3228247

[6] Suki B, Lutchen KR, Ingenito EP. On the progressive nature of emphysema: roles of proteases, inflammation, and mechanical forces. Am J Respir Crit Care Med 2003;168(5):516–521. https://doi.org/10.1164/rccm.200208-908PP 12941655 10.1164/rccm.200208-908PP

[7] Tanabe N, Muro S, Sato S, Tanaka S, Oguma T, et al Longitudinal Study of Spatially Heterogeneous Emphysema Progression in Current Smokers with Chronic Obstructive Pulmonary Disease. PLoS ONE 2012;7(9):e44993 https://doi.org/10.1371/journal.pone.0044993 23028728 10.1371/journal.pone.0044993 PMC3445600

[8] Pauwels RA, Buist AS, Calverley PM, Jenkins CR, Hurd SS, Gold Scientific Committee. Global strategy for the diagnosis, management, and prevention of chronic obstructive pulmonary disease: NHLBI/WHO Global Initiative for Chronic Obstructive Lung Disease (GOLD) Workshop summary. Am J Respir Crit Care Med. 2001;163(5):1256–76. https://doi.org/10.1164/ajrccm.163.5.2101039 11316667 10.1164/ajrccm.163.5.2101039

[9] Roach ES, Gomez MR, Northrup H. Tuberous sclerosis complex consensus conference: revised clinical diagnostic criteria. J Child Neurol 1998;13(12):624–8. https://doi.org/10.1177/088307389801301206 9881533 10.1177/088307389801301206

[10] Gunji Y, Akiyoshi T, Sato T, Kurihara M, Tominaga S, Takahashi K, et al Mutations of the Birt–Hogg–Dubé gene in patients with multiple lung cysts and recurrent pneumothorax. J Med Genet 2007;44(9):588–93. https://doi.org/10.1136/jmg.2007.049874 17496196 10.1136/jmg.2007.049874 PMC2597956

[11] Ohara T, Hirai T, Sato S, Sato A, Nishioka M, Muro S, et al Comparison of airway dimensions in different anatomic locations on chest CT in patients with COPD. Respirology 2006;11(5):579–85. https://doi.org/10.1111/j.1440-1843.2006.00899.x 16916330 10.1111/j.1440-1843.2006.00899.x

[12] Tonan T, Fujimoto K, Qayyum A, Kawaguchi T, Kawaguchi A, Nakashima O, et al Quantification of hepatic iron concentration in chronic viral hepatitis: usefulness of T2-weighted single-shot spin-echo echo-planar MR imaging. PLoS One.2012;7:e33868 https://doi.org/0.1371/journal.pone.0033868 22439008 10.1371/journal.pone.0033868 PMC3306306

[13] Snider GL, Kleinerman J, Thurlbeck WM, Bengali ZH. The definition of emphysema. Report of a National Heart, Lung, and Blood Institute, Division of Lung Diseases workshop. Am Rev Respir Dis. 1985;132(1):182–5. https://doi.org/10.1164/arrd.1985.132.1.182 4014865 10.1164/arrd.1985.132.1.182

[14] Sharafkhaneh A, Hanania NA, Kim V. Pathogenesis of emphysema: from the bench to the bedside. Proc Am Thorac Soc. 2008;5(4):475–7. https://doi.org/10.1513/pats.200708-126ET 18453358 10.1513/pats.200708-126ET PMC2645322

[15] Zhe X, Yang Y, Jakkaraju S, Schuger L. Tissue inhibitor of metalloproteinase-3 downregulation in lymphangioleiomyomatosis: potential consequence of abnormal serum response factor expression. Am J Respir Cell Mol Biol 2003;28(4):504–11. https://doi.org/10.1165/rcmb.2002-0124OC 12654640 10.1165/rcmb.2002-0124OC

[16] Hayashi T, Fleming MV, Stetler-Stevenson WG, Liotta LA, Moss J, Ferrans VJ, et al Immunohistochemical study of matrix metalloproteinases (MMPs) and their tissue inhibitors (TIMPs) in pulmonary lymphangioleiomyomatosis (LAM). Hum Pathol 1997; 28(9):1071–8. 9308732 10.1016/s0046-8177(97)90061-7

[17] Matsui K, Takeda K, Yu ZX, Travis WD, Moss J, Ferrans VJ. Role for activation of matrix metalloproteinases in the pathogenesis of pulmonary lymphangioleiomyomatosis. Arch Pathol Lab Med 2000;124(2):267–75. https://doi.org/10.1043/0003-9985(2000)124<0267:RFAOMM>2.0.CO;2 10656737 10.5858/2000-124-0267-RFAOMM

[18] Travis WD, Usuki J, Horiba K, Ferrans VJ. Histopathologic studies on lymphangioleiomyomatosis In: Moss J, ed. LAM and other diseases characterized by smooth muscle proliferation. Lung biology in health and disease, vol 131 New York, NY: Marcel Dekker, 1999;171–217

[19] Pallisa E, Sanz P, Roman A, Majó J, Andreu J, Cáceres J. Lymphangioleiomyomatosis: pulmonary and abdominal findings with pathologic correlation. Radiographics 2002;22:S185–98. https://doi.org/10.1148/radiographics.22.suppl_1.g02oc13s185 12376610 10.1148/radiographics.22.suppl_1.g02oc13s185

[20] Lenoir S, Grenier P, Brauner MW, Frija J, Remy-Jardin M, Revel D, et al Pulmonary lymphangiomyomatosis and tuberous sclerosis: comparison of radiographic and thin-section CT findings. Radiology 1990;175(2):329–34. https://doi.org/10.1148/radiology.175.2.2326456 2326456 10.1148/radiology.175.2.2326456

[21] Sullivan EJ. Lymphangioleiomyomatosis: a review. Chest 1998;114(6):1689–703. 9872207 10.1378/chest.114.6.1689

[22] Birt AR, Hogg GR, Dubé WJ. Hereditary multiple fibrofolliculomas with trichodiscomas and acrochordons. Arch Dermatol 1977;113(12):1674–7. 596896

[23] Schmidt LS, Nickerson ML, Warren MB, Glenn GM, Toro JR, Merino MJ, et al Germline BHD-mutation spectrum and phenotype analysis of a large cohort of families with Birt-Hogg-Dubé syndrome. Am J Hum Genet 2005;76(6):1023–33. https://doi.org/10.1086/430842 15852235 10.1086/430842 PMC1196440

[24] Nickerson ML, Warren MB, Toro JR, Matrosova V, Glenn G, Turner ML, et al Mutations in a novel gene lead to kidney tumors, lung wall defects, and benign tumors of the hair follicle in patients with the Birt-Hogg-Dubé syndrome. Cancer Cell 2002;2(2):157–64. 12204536 10.1016/s1535-6108(02)00104-6

[25] Graham RB, Nolasco M, Peterlin B, Garcia CK. Nonsense mutations in folliculin presenting as isolated familial spontaneous pneumothorax in adults. Am J Respir Crit Care Med 2005;172(1):39–44. https://doi.org/10.1164/rccm.200501-143OC 15805188 10.1164/rccm.200501-143OC

[26] Painter JN, Tapanainen H, Somer M, Tukiainen P, Aittomaki K. A 4-bp deletion in the Birt-Hogg-Dube gene (FLCN) causes dominantly inherited spontaneous pneumothorax. Am J Hum Genet 2005;76(3):522–7. https://doi.org/10.1086/428455 15657874 10.1086/428455 PMC1196403

[27] Furuya M, Tanaka R, Koga S, Yatabe Y, Gotoda H, Takagi S, et al Pulmonary cysts of Birt–Hogg–Dube syndrome: a clinicopathologic and immunohistochemical study of 9 families. Am J Surg Pathol 2012; 36(4); 589–600. https://doi.org/10.1097/PAS.0b013e3182475240 22441547 10.1097/PAS.0b013e3182475240

[28] Kumasaka T, Hayashi T, Mitani K, Kataoka H, Kikkawa M, Tobino K, et al Characterization of pulmonary cysts in Birt-Hogg-Dubé syndrome: histopathological and morphometric analysis of 229 pulmonary cysts from 50 unrelated patients. Histopathology 2014;65(1):100–10. https://doi.org/10.1111/his.12368 24393238 10.1111/his.12368 PMC4237186

[29] Goncharova EA, Goncharov DA, James ML, Atochina-Vasserman EN, Stepanova V, Hong SB, et al Folliculin Controls Lung Alveolar Enlargement and Epithelial Cell Survival through E-cadherin, LKB1 and AMPK. Cell Rep. 2014;7(2):412–23. https://doi.org/10.1016/j.celrep.2014.03.025 24726356 10.1016/j.celrep.2014.03.025 PMC4034569

