# Supplementary figures and images for: Difference of the progression of pulmonary cysts assessed by computed tomography among COPD, lymphangioleiomyomatosis, and Birt-Hogg-Dubé syndrome
Source: PLoS One. 2017 Dec 8;12(12):e0188771. doi: 10.1371/journal.pone.0188771 (PMC5722335; doi:10.1371/journal.pone.0188771)

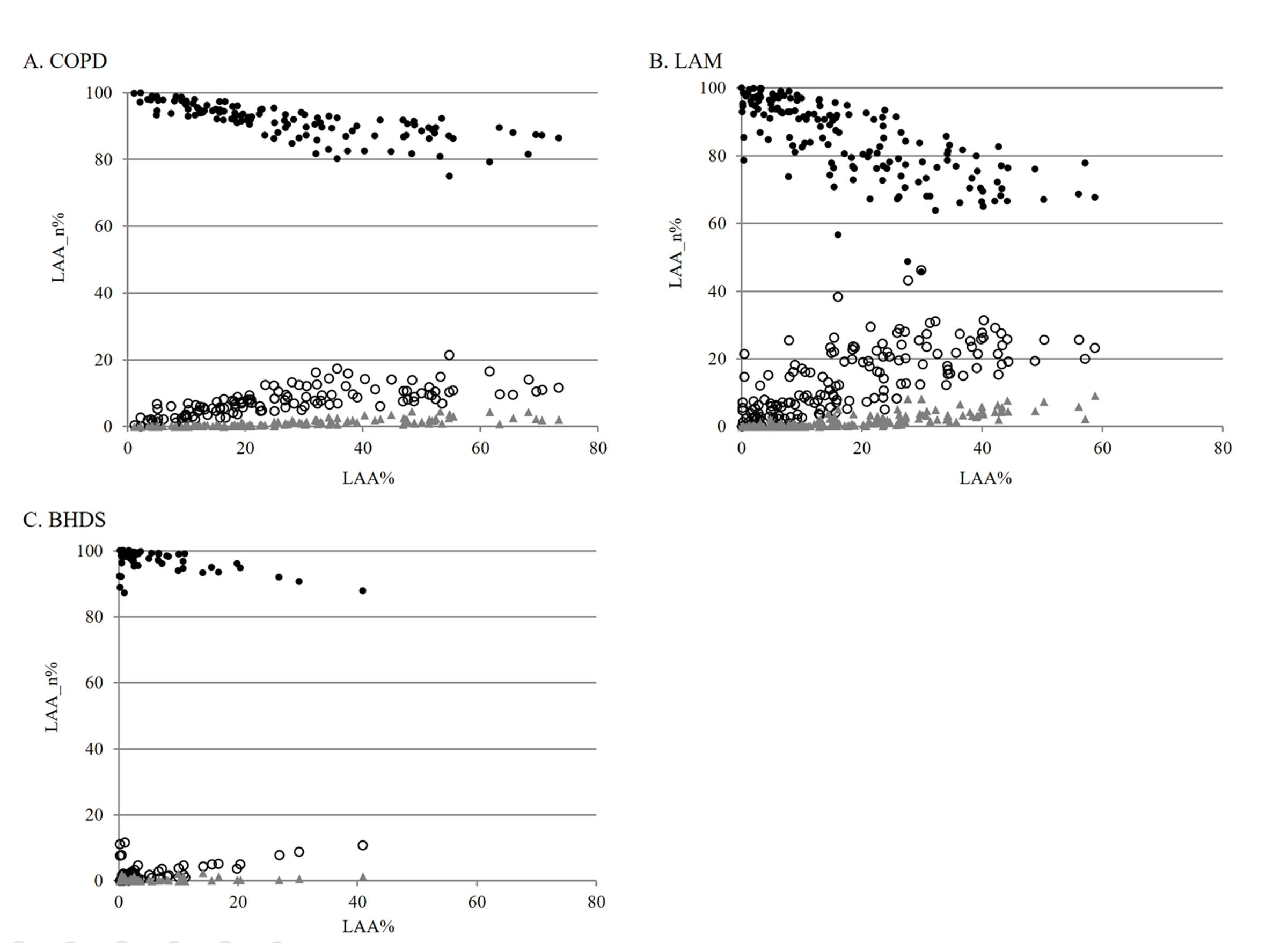

Supplement: S1 Fig — LAA_n% means the ratio (%) of each size-catergorized LAA_n {i.e., small (●), medium (○), and large (▲)} to the entire LAA_n of the CT image. In LAM, the increase of medium LAA_n% stands out more than other diseases. BHDS: Birt-Hogg-Dubé syndrome; COPD: chronic obstructive pulmonary disease; LAA%: percentage of lung field occupied by low attenuation areas; LAA_n: number of LAA cluster; LAM: lymphangioleiomyomatosis. (TIF) [file pone.0188771.s001.tif]

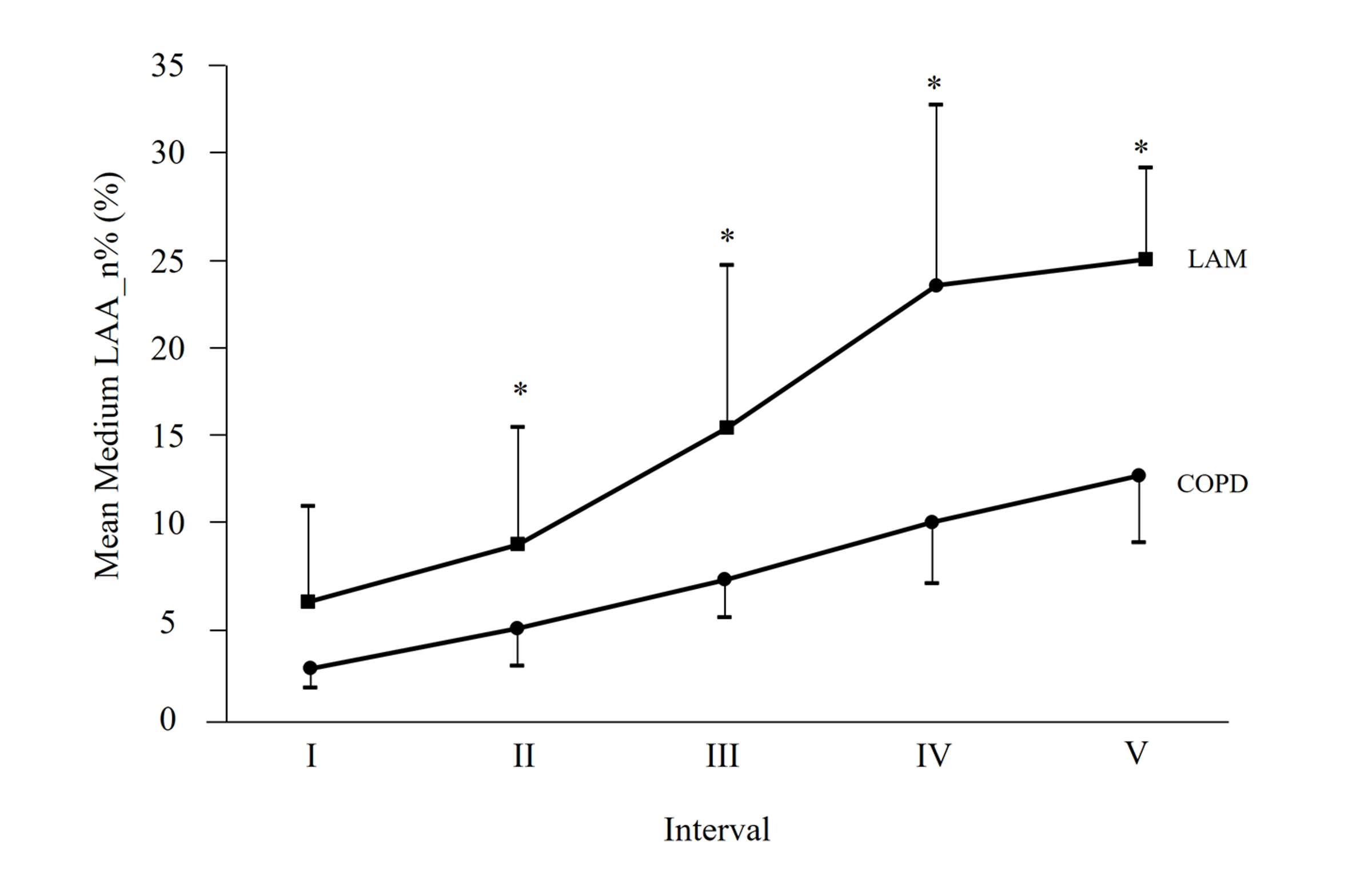

Supplement: S2 Fig — LAM is significantly higher than COPD in the interval II to V. The standard deviation values are shown with short horizontal lines. COPD: chronic obstructive pulmonary disease; LAA%: percentage of lung field occupied by low attenuation areas; LAA_n: number of LAA cluster; LAA_n%: the ratio (%) of each size-catergorized LAA_n {i.e., small (●), medium (○), and large (▲)} to the entire LAA_n of the CT image; LAM: lymphangioleiomyomatosis * p < 0.01. (TIF) [file pone.0188771.s002.tif]
